# Long-term neurocognitive functioning of children treated with propranolol or atenolol for infantile hemangioma

**DOI:** 10.1007/s00431-022-04674-7

**Published:** 2022-12-07

**Authors:** Mireille M. Hermans, André B. Rietman, Renske Schappin, Peter C. J. de Laat, Elodie J. Mendels, Johannes M. P. J. Breur, Hester R. Langeveld, Saskia N. de Wildt, Corstiaan C. Breugem, Marlies de Graaf, Martine F. Raphael, Suzanne G. M. A. Pasmans

**Affiliations:** 1 Department of Dermatology, Erasmus MC Sophia Children’s Hospital, University Medical Center Rotterdam, Center of Pediatric Dermatology, Center of Rare Skin Diseases, Vascular Anomaly Center Erasmus MC Rotterdam, member of the ERN-SKIN-Mosaic group and ERN-VASCERN-VASCA group, Rotterdam, Netherlands; 2 Department of Child and Adolescent Psychology/Psychiatry, Erasmus MC Sophia Children’s Hospital, University Medical Center Rotterdam, Rotterdam, Netherlands; 3 Department of Surgery, Wilhelmina Children’s Hospital, University Medical Center Utrecht, Utrecht, Netherlands; 4 Department of Pediatrics(-Hemato-Oncology), Center of Rare Skin Diseases, Vascular Anomaly Center Erasmus MC Rotterdam, member of the ERN-SKIN-Mosaic group and ERN-VASCERN-VASCA group, Erasmus MC Sophia Children’s Hospital, University Medical Center Rotterdam, Rotterdam, Netherlands; 5 Department of Pediatric Cardiology, Wilhelmina Children’s Hospital, University Medical Center Utrecht, Utrecht, Netherlands; 6 Department of Intensive Care and Pediatric Surgery, Center of Rare Skin Diseases, Vascular Anomaly Center Erasmus MC Rotterdam, member of the ERN-SKIN-Mosaic group and ERN-VASCERN-VASCA group, Erasmus MC Sophia Children’s Hospital, University Medical Center Rotterdam, Rotterdam, Netherlands; 7 Department of Pharmacology and Toxicology, Radboud Institute for Health Sciences, Radboud University Medical Center, Nijmegen, Netherlands; 8 Department of Plastic Surgery, UMC Utrecht Center for Vascular Anomalies, Wilhelmina Children’s Hospital, University Medical Center Utrecht, Utrecht, Netherlands; 9 Department of Plastic, Reconstructive and Hand Surgery, UMC Location University of Amsterdam, Amsterdam, Netherlands; 10 Department of Dermatology, UMC Utrecht Center for Vascular Anomalies, Wilhelmina Children’s Hospital, University Medical Center Utrecht, Utrecht, Netherlands; 11 Department Emma Children’s Hospital, UMC Location University of Amsterdam, Amsterdam, Netherlands

**Keywords:** Capillary hemangioma, Vascular tissue neoplasms, Vascular malformations, Adrenergic beta-antagonists, Long-term adverse effects, Neuropsychological tests, Wechsler scales, Neurocognitive disorders, Cognition, Executive function, Child, Infant

## Abstract

The purpose of this study was to compare long-term neurocognitive functioning (working memory, processing speed, and attention) between children who had been treated with either propranolol or atenolol for infantile hemangioma during infancy. All eligible children (*n* = 158) aged 6 years or older and treated with propranolol or atenolol as infants were invited to participate in this two-center cross-sectional study. The primary outcome was the Wechsler Intelligence Scale for Children-V Cognitive Proficiency Index (CPI), a measure of working memory, processing speed, and attention. Secondary outcomes were general intelligence, auditory, visuospatial, and narrative memory, as well as executive functioning and sleep. A total of 105 children, of whom 36 had been treated with propranolol (age 6.0–11.8 years, follow-up time 1.6–9.7 years, 19% male) and 69 had been treated with atenolol (age 6.9–9.7 years, follow-up time 4.5–8.4 years, 19% male), were analyzed. The CPI and other neurocognitive outcomes did not differ between the propranolol and atenolol groups and were in line with general population test norms. Post hoc analyses revealed lower CPI scores for males, both compared to participating females (10.3 IQ points, medium effect size) and compared to matched test norms (12.4 IQ points, medium effect size).

*Conclusions*: Long-term neurocognitive functioning did not differ between children treated with propranolol and those treated with atenolol for IH. Overall, propranolol and atenolol appear to be safe treatments for IH regarding long-term neurocognitive functioning. The substantially lower CPI scores in males warrant further investigation.

*Trial registration*: Netherlands Trial Register, NL7703 https://www.trialregister.nl/trial/7703

**Table:** 

**What is Known:**
• *Infants with infantile hemangioma are effectively treated with propranolol or atenolol.*
• *Parents and professionals are concerned about long-term neurocognitive effects.*
**What is New:**
• *No long-term (*≥ *6 years) differences in neurocognitive functioning were found between children treated with propranolol or atenolol.*
• *Males treated with beta-blockers had substantially lower IQ scores than treated females and males from the general population, which is a matter of concern and should be considered when evaluating the risk/benefit ratio in less severe forms of infantile hemangioma.*

**Supplementary Information:**

The online version contains supplementary material available at 10.1007/s00431-022-04674-7.

## Introduction

Infantile hemangiomas (IH) are the most common vascular tumors of childhood, with estimated incidences ranging from 2.0 to 4.5% [1, 2]. A substantial proportion of otherwise healthy infants with IH requires treatment with beta-blockers to prevent or treat complications, such as ulceration, functional impairment, or disfigurement [3]. Concerns have been raised about the long-term impact of propranolol, a lipophilic beta-blocker, due to possible treatment effects on the central nervous system (CNS) at a vulnerable age [4]. Previous clinical studies have shown that atenolol, a hydrophilic beta-blocker, is as effective as propranolol but seems to be associated with fewer CNS effects during IH treatment [5, 6]. Since 2014, propranolol has been the only worldwide approved beta-blocker to treat IH. Atenolol has been frequently prescribed for IH, though off-label [7, 8].

To date, no long-term neurocognitive problems in children treated with beta-blockers for IH have been reported [9–12]. However, the generalizability of previous studies was limited due to small sample sizes (*n* = 23 [11] and *n* = 27 [12]). Furthermore, previously used outcome measures such as general intelligence or broad neurodevelopmental milestones are not sensitive to subtle deviations in complex neurocognitive functions, e.g., working memory, processing speed, and attention [9, 10]. Also, previous research did not compare the long-term effects between propranolol and a hydrophilic beta-blocker, such as atenolol. Therefore, the aim of this study was to investigate and compare long-term neurocognitive outcomes (i.e., working memory, processing speed, and attention) in school-aged children who had been treated with either propranolol or atenolol for IH during infancy.

## Materials and methods

### Design

This two-center cross-sectional study was conducted at the vascular anomaly centers of the Erasmus MC, University Medical Center Rotterdam (Erasmus MC, Rotterdam, the Netherlands), and the University Medical Center Utrecht (UMCU, Utrecht, the Netherlands) [13]. Both centers introduced propranolol treatment in 2008. UMCU switched to atenolol treatment in 2009 and Erasmus MC switched to atenolol treatment in 2013, based on positive clinical experience and before propranolol was globally approved [14]. This enabled us to study an internationally unique cohort of school-aged children, who had received either propranolol or atenolol, independent of their disease characteristics.

Children were assessed during an outpatient visit, consisting of a neuropsychological assessment by a psychologist (MH), a standard physical examination by a pediatrician (PdL, JB, MR), and a dermatological examination by a pediatric dermatologist (MdG, SP). Children’s parents completed questionnaires about the child’s psychological, neurocognitive, and physical development. Information on IH treatment was retrieved from medical records.

### Participants

Prior to recruitment, we screened records of all patients born between 2008 and 2014 who were treated for IH at either center to identify any eligible children. Children were actively recruited between April and December 2019; the last recruited child was assessed in February 2020.

The inclusion criteria were (1) age ≥ 6 years upon participation in neuropsychological assessment; (2) IH previously treated with either oral propranolol at ≥ 2 mg/kg/day or oral atenolol at ≥ 1 mg/kg/day; (3) treatment duration ≥ 6 months; (4) treatment initiated before the age of 1 year; (5) IQ estimated > 55 (no moderate to severe intellectual disability); and (6) child and parent(s)/legal guardian(s) having sufficient comprehension of the Dutch language to understand study materials. The exclusion criteria were (1) prematurity < 37 weeks of gestation; (2) low birth weight (< 2.5 SD for gestational age); (3) complicated neonatal period with hospitalization; (4) suspected PHACE syndrome; (5) other treatment than oral propranolol or atenolol for IH (such as other oral beta-blockers, oral corticosteroids, vincristine, interferon alpha, topical beta-blockers, intralesional corticosteroids, imiquimod, rapamycin, laser, surgery, and cryotherapy); (6) documented psychological or neurocognitive problems before starting beta-blockers; (7) medication that could negatively affect psychological or neurocognitive functioning (including multiple general anesthesia); (8) genetic syndromes known to affect cognitive performance; (9) concomitant or successive use of propranolol and atenolol; and (10) participation in a study or compassionate use program with ID V0400SB.

This study was exempt from the Dutch Medical Research Involving Human Subjects Act according to the institutional review boards of Erasmus MC (MEC-2019–0268) and UMCU (19–115/C). All parent(s)/legal guardian(s) provided written informed consent.

### Measurements

We included those measures of neurocognitive functions that have been documented to be affected by beta-blockers [4]. All measures are standardized for children aged 6 to 12 years, have age-corrected normed scores based on the general Dutch population, and have sufficient psychometric properties [15–18].

The primary outcome measure was the Cognitive Proficiency Index (CPI), a subscale of the Wechsler Intelligence Scale for Children-V, Dutch version (WISC-V-NL). The CPI comprises four subtests that measure working memory and processing speed. Attention may be inferred based on demands required to complete these subtests. The CPI composite score is more reliable than the individual subtests. CPI standardized scores have a mean of 100 and a standard deviation of 15 points [15].

Secondary outcomes were general intelligence (full-scale intelligence quotient (FSIQ) and general ability index (GAI) of the WISC-V-NL), as well as auditory, visuospatial, and narrative memory. Auditory memory was measured with the Rey Auditory Verbal Learning Test (RAVLT). This included immediate recall (auditory working memory and attention) and delayed recall based on scores at immediate recall (long-term auditory memory). Raw scores were age-corrected and converted into Z-scores (mean 0, standard deviation 1) [16]. Visuospatial memory was evaluated with the Dutch version of the Developmental Neuropsychological Assessment-II (NEPSY-II-NL), subtest Memory for Designs (MD) and Memory for Designs Delayed (MDD). MD assesses spatial memory for novel visual material, while MDD assesses long-term visuospatial memory. Verbal narrative memory was assessed with the NEPSY-II-NL subtest Narrative Memory. Total scores of all NEPSY-II-NL subtests were converted into age-corrected percentiles [17]. Percentiles ≤ 10 were considered to be in the clinical range.

Parents reported on their child’s executive functioning in daily life and sleep habits. Executive functioning was assessed with the Behavioral Rating Inventory of Executive Function (BRIEF), which resulted in T-scores (mean 50, standard deviation 10) [18]. Sleep habits were assessed with the Child Sleep Habits Questionnaire (CSHQ) [19]. The CSHQ allows for a total score, which reflects the major sleep disorders in children aged 4 to 11 years old. The mother’s highest completed education level was used for its association with socioeconomic status and parent intelligence and was categorized according to the International Standard Classification of Education (ISCED) [20].

We retrieved the following information from patient records: the child’s sex, age at treatment initiation (months), treatment duration (months, excluding temporary treatment interruptions), maximum dose (mg/kg/day), average dose (mg/kg/day), and cumulative dose (total exposure corrected for weight, mg/kg).

One certified psychologist performed all neuropsychological assessments, blinded to the type of beta-blocker treatment the child had received as an infant and the treatment practices in both centers.

### Data analysis

All test assumptions were checked prior to data analysis. This included tests for normality of continuous data, using inspection of plots, means and medians, kurtosis and skewness, and Shapiro–Wilk testing.

#### Comparisons between propranolol and atenolol groups

We used independent samples *t* tests to analyze differences in our primary outcome (CPI) between children treated with propranolol and those treated with atenolol. A multivariable linear regression, with CPI as the dependent variable and beta-blocker type as predictor, was performed controlling for the child’s sex, the mother’s education level, the child’s age at treatment initiation, treatment duration, and cumulative dose. For normally distributed secondary outcomes at interval level, we used the same procedure as for the CPI. Non-normally distributed or ordinal outcomes were analyzed with Mann–Whitney *U* tests and multivariable linear regression. Dichotomous variables were analyzed with Fisher’s exact tests and logistic linear regression.

#### Comparisons to general population norms

Differences between sample scores and general population norms were analyzed using one-sample *t* tests or one-sample Wilcoxon signed rank tests for skewed data. We compared dichotomous data with expected proportions using chi-square tests. If any differences were observed between the beta-blocker groups, analyses were performed independently for each beta-blocker group.

All data were entered into an online OpenClinica 3.12.2. database and analyzed by authors MH, AR, and RS using SPSS 25.0. As missing data were rare (< 5% of data), we used complete case analysis. A two-sided *p* < 0.05 was considered statistically significant in primary analyses. Accounting for multiple comparisons, a two-sided *p* < 0.002 was considered statistically significant in secondary analyses (Dunn–Šidák correction). Effect sizes were calculated corresponding to each statistical method and interpreted according to Cohen’s guidelines [21].

## Results

### Participant characteristics

**Fig. 1:**
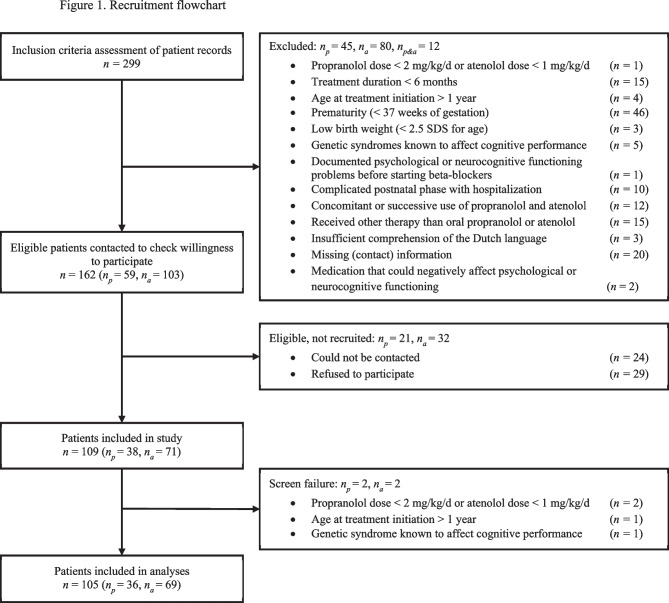
Recruitment flowchart. Abbreviations: *n*_*p*_, number of patients treated with propranolol; *n*_*a*_, number of patients treated with atenolol; *n*_*p&a*_, number of patients treated with both propranolol and atenolol [13]

Figure 1 shows the flow chart of the inclusion process. Consent to participate was obtained for 109 of the 162 potentially eligible children. After inclusion, 4 children were considered screen failures, resulting in a final sample of 105 children (66% of the 158 eligible children; 78% of the 134 successfully contacted eligible children), consisting of 36 children treated with propranolol and 69 children treated with atenolol.

Participant characteristics are described in Table 1. There was a female predominance (81%), consistent with the literature [22]. The sex ratio was the same in both beta-blocker groups. As propranolol was initiated before atenolol in both treatment centers, children treated with propranolol were significantly older than children treated with atenolol. All outcomes were age-corrected; thus, this difference did not affect our results. The prevalence of attention-deficit/hyperactivity disorder (ADHD) (5.7%) was in line with the estimated population prevalence among children and adolescents (5.9–7.1%) [23]. Four children (3.8%) were treated with methylphenidate. In accordance with test criteria of the WISC-V-NL, children with ADHD (with or without methylphenidate) were included in the study [15].

**Table 1:** Participant characteristics

	All (*n* = 105)	Propranolol (*n* = 36)	Atenolol (*n* = 69)	*p* value
**Demographics**				
Child age, years				
Median (*IQR*)	7.4 (6.7–8.5)	8.0 (7.3–8.8)	7.1 (6.4–8.1)	< 0.001
Range	6.0–11.8	6.4–11.8	6.0–9.7	
Child sex, *n* (%)				
Female	85 (81)	29 (81)	56 (81)	> 0.99
Male	20 (19)	7 (19)	13 (19)	
Child migration background^a^, *n* (%)				
Yes	10 (10)	0 (0.0)	10 (14)	0.01
No	94 (90)	36 (100)	58 (84)	
Unknown	1 (1.0)	0 (0.0)	1 (1.4)	
Education mother, *n* (%)				
Low	14 (13)	6 (17)	8 (12)	0.89
Average	28 (27)	8 (22)	20 (29)	
High	62 (59)	22 (61)	40 (58)	
Unknown	1 (1.0)	0 (0.0)	1 (1.4)	
Home language, *n* (%)				
Dutch	97 (92)	35 (97)	62 (90)	0.37
Other	2 (2.0)	0 (0.0)	2 (2.9)	
Multilingual	6 (5.7)	1 (2.8)	5 (7.2)	
Confirmed diagnosis, *n* (%)				
Attention-deficit/hyperactivity disorder	6 (5.7)	3 (8.3)	3 (4.3)	
**Clinical information**				
Location of IH^b^, *n* (%)				
Head and neck	84 (80)	24 (67)	60 (87)	0.02
Trunk	13 (12)	6 (17)	7 (10)	0.36
Genital area	13 (12)	7 (19)	6 (8.7)	0.13
Extremities	7 (6.7)	3 (8.3)	4 (5.8)	0.69
Ulcerated IH, *n* (%)				
Yes	29 (28)	13 (36)	16 (23)	0.16
No	76 (72)	23 (64)	53 (77)	
Treatment center, *n* (%)				
Erasmus MC	34 (32)	31 (86)	3 (4.3)	< 0.001
UMCU	71 (68)	5 (14)	66 (96)	
Age at treatment initiation, months				
Median (*IQR*)	3.5 (2.2–5.1)	3.6 (2.2–5.3)	3.4 (2.2–5.0)	0.58
Range	0.92–11.4	1.64–11.4	0.92–10.9	
Treatment duration, months				
Median (*IQR*)	13.8 (10.9–19.4)	18.6 (12.5–22.7)	13.0 (10.4–15.8)	0.001
Range	6.41–62.7	9.13–62.7	6.41–56.8	
Average dose, mg/kg/day				
Median (*IQR*)	1.2 (1.0–1.8)	1.9 (1.8–2.0)	1.0 (1.0–1.2)	< 0.001
Range	0.8–2.5	1.4–2.5	0.8–2.0	
Peak dose, mg/kg/day				
Median (*IQR*)	1.6 (1.0–2.1)	2.1 (2.0–2.3)	1.0 (1.0–1.6)	< 0.001
Range	1.0–14.0	1.9–14.0	1.0–3.0	
Cumulative dose, mg/kg				
Median (*IQR*)	577.4 (387.2–881.7)	1122.7 (718.6–1282.3)	418.7 (310.0–619.7)	< 0.001
Range	186.6–3544	494.1–3544	186.6–2206	
Follow-up time^c^, years				
Median (*IQR*)	5.9 (5.2–6.5)	6.2 (5.6–6.6)	5.7 (5.1–6.2)	0.13
Range	1.6–9.7	1.6–9.7	4.5–8.4	

*p* values indicate differences in participant characteristics between propranolol and atenolol group Continuous variables were not normally distributed and analyzed with a Mann–Whitney *U* test. Dichotomous variables were analyzed with a Fisher’s exact test

^a^Child migration background, categorized as “yes” = one or both parents born abroad or “no” = both parents born in the Netherlands

^b^A total of 105 patients had a total of 128 IH. The variable “location of IH” represents the number of children with at least one infantile hemangioma at each region

^c^Follow-up time: time interval between cessation of beta-blocker treatment and neuropsychological assessment

Corresponding to the transition to atenolol at UMCU in 2009 and at Erasmus MC in 2013, almost all atenolol-treated children were treated at UMCU, and most propranolol-treated children were treated at Erasmus MC. To avoid multicollinearity, we did not control the analyses for treatment center.

Given the standard maintenance dose of 2 mg/kg/day for propranolol and 1 mg/kg/day for atenolol, significant differences in average, cumulative, and peak dose between children treated with propranolol and atenolol were as expected. Treatment duration differed significantly between both beta-blocker groups; the median duration was almost 6 months longer for children treated with propranolol. Given that all IH were treated until sufficient clinical improvement was achieved, this difference in treatment duration may reflect higher severity of IH treated with propranolol compared to IH treated with atenolol.

### Comparison between propranolol and atenolol

The primary outcome measure CPI was normally distributed and did not differ between children treated with propranolol (*M* = 98.9, *SD* = 17.4) and children treated with atenolol (*M* = 101.6, *SD* = 12.8; *p* = 0.38) (Table 2). Similarly, analysis corrected for confounders showed no significant effect of beta-blocker type on CPI scores (*p* = 0.81). None of the secondary outcomes differed between the two groups.

**Table 2:** Analyses of the difference in neurocognitive functioning between children treated with propranolol and atenolol for IH

	Descriptive statistics		Univariate analyses for the comparison propranolol vs. atenolol		Multivariate analyses for the comparison propranolol vs. atenolol, adjusted for covariates^1^			
	Propranolol (*n* = 36)	Atenolol (*n* = 69)	*p* value	Effect size	*B* or *OR* (95% CI)		*p* value	Effect size^d^ *f*^2^
Intelligence (WISC-V-NL), *M* (*SD*)								
** Cognitive Proficiency Index**^**g**^	98.9 (17.4)	101.6 (12.8)	0.38	0.20^a^	*B* =	1.0 (− 7.9–9.9)	0.81	0.00
General ability index	101.2 (12.6)	101.5 (11.8)	0.92	0.00^a^	*B* =	2.4 (− 5.1–9.9)	0.52	0.00
Full-scale IQ	100.7 (14.0)	100.7 (11.6)	0.99	0.00^a^	*B* =	1.0 (− 6.6–8.7)	0.79	0.00
Visual Spatial Memory (NEPSY-II-NL), *n* (%)								
Immediate recall								
Clinical range (pct ≤ 10)	5 (14)	10 (14)	> 0.99	0.01^b^	*OR* =	0.5 (0.1–3.0)^e^	0.42	N.A
Non-clinical range (pct > 10)	31 (86)	59 (86)						
Delayed recall								
Clinical range (pct ≤ 10)	7 (19)	8 (12)	0.38	0.11^b^	*OR* =	0.3 (0.0–1.9)^e^	0.20	N.A
Non-clinical range (pct > 10)	29 (81)	61 (88)						
Narrative Memory (NEPSY-II-NL), *n* (%)								
Clinical range (pct ≤ 10)	2 (5.6)	8 (12)	0.49	0.10^b^	*OR* =	0.8 (0.1–11.1)^e^	0.84	N.A
Non-clinical range (pct > 10)	34 (94)	61 (88)						
Auditory Memory (RAVLT)^h^, *M* (*SD*)								
Immediate recall	0.0 (1.2)	− 0.4 (0.9)	0.13	0.38^a^	*B* =	− 0.7 (− 1.3– − 0.0)	0.036	0.04
Delayed recall	− 0.1 (1.3)	− 0.4 (0.9)	0.18	0.27^a^	*B* =	− 0.7 (− 1.3– − 0.0)	0.039	0.04
Executive Functioning (BRIEF)^i^, *Mdn* (*IQR*)								
Behavioral regulation index	43.0 (37.0–55.5)	38.0 (33.0–50.0)	0.13	0.15^c^	*B* =	− 4.4 (− 11.3–2.6)	0.21	0.02
Metacognition index	42.5 (36.3–52.0)	39.0 (33.0–50.0)	0.19	0.13^c^	*B* =	− 4.3 (− 11.2–2.6)	0.22	0.01
Total score	44.0 (35.5–51.8)	38.0 (31.0–48.0)	0.14	0.14^c^	*B* =	− 4.6 (− 12.0–2.7)	0.21	0.02
Sleep behavior (CSHQ)^j^, *Mdn* (*IQR*)								
Total score	41.0 (37.3–45.8)	42.0 (37.0–46.3)	0.55	0.06^c^	*B* =	2.5 (− 1.5–6.4)	0.22	0.02

*N.A.*, not applicable. Continuous variables are analyzed with independent samples *t* tests, Mann–Whitney *U* tests, and multivariable linear regression (for covariate-adjusted analyses). Dichotomous variables are analyzed with Fisher’s exact test and logistic linear regression (for covariate-adjusted analyses). *p* < 0.05 is considered statistically significant for primary outcome analyses (CPI). *p* < 0.002 is considered statistically significant for secondary outcome analyses (Dunn–Šidák correction). Effect sizes are were calculated corresponding to each statistical method and interpreted according to Cohen’s guidelines [21]

^a^Effect size Cohen’s *d*, small = 0.2, medium = 0.5, large = 0.8

^b^Effect size phi, small = 0.1, medium = 0.3, large = 0.5

^c^Effect size Pearson’s *r*, small = 0.1, medium = 0.3, large = 0.5

^d^Effect size *f*^2^, small = 0.02, medium = 0.15, large = 0.35

^e^Odds Ratio (OR), small = 1.68, medium = 3.47, large = 6.71 [29]

^f^Corrected for socioeconomic status, child sex, cumulative dose (mg/kg), treatment duration (months), and age at treatment initiation (months)

^g^One atenolol-treated child had a missing CPI score, *n* = 104 (propranolol *n* = 36; atenolol *n* = 68)

^h^Results excluding two atenolol-treated outliers that deviated more than 3 SD from sample average due to unreliable assessment, *n* = 103 (propranolol *n* = 36; atenolol *n* = 67)

^i^Two atenolol-treated children had missing BRIEF scores, *n* = 103 (propranolol *n* = 36; atenolol *n* = 67)

^j^Four propranolol-treated children and two atenolol-treated children had missing CSHQ scores, *n* = 98 (propranolol *n* = 32; atenolol *n* = 66)

### Comparison between beta-blockers and norm data

The sample mean of the primary outcome CPI (*M* = 100.7, *SD* = 14.5) was not significantly different from norm data (*M* = 100, *SD* = 15; *p* = 0.64); the effect size was small (Table 3). Scores on all secondary outcomes, with the exception of the BRIEF, did not differ from norm scores. Parents of children treated with beta-blockers scored significantly lower (i.e., better) than test norms on the BRIEF, with a large effect size.

**Table 3:** Univariate analyses of the difference in neurocognitive functioning between children treated with beta-blockers for IH and normed scores based on the general Dutch population

	All (*n* = 105)	Norm scores	*p* value	Effect size
Intelligence (WISC-V-NL), *M* (*SD*)				
** Cognitive Proficiency Index**^**d**^	100.7 (14.5)	100 (15)	0.64	0.07^a^
General ability index	101.4 (12.0)	100 (15)	0.24	0.07^a^
Full-scale IQ	100.7 (12.4)	100 (15)	0.56	0.07^a^
Visual Spatial Memory (NEPSY-II-NL), *n* (%)				
Immediate recall				
Clinical range (pct ≤ 10)	15 (14)	10%	0.14	0.14^b^
Non-clinical range (pct > 10)	90 (86)	90%		
Delayed recall				
Clinical range (pct ≤ 10)	15 (14)	10%	0.14	0.14^b^
Non-clinical range (pct > 10)	90 (86)	90%		
Narrative Memory (NEPSY-II-NL), *n* (%)				
Clinical range (pct ≤ 10)	10 (10)	10%	0.87	0.02^b^
Non-clinical range (pct > 10)	95 (90)	90%		
Auditory Memory (RAVLT)^e^, *M* (*SD*)				
Immediate recall	− 0.2 (1.1)	0 (1.0)	0.026	0.23^a^
Delayed recall	− 0.3 (1.1)	0 (1.0)	0.013	0.26^a^
Executive Functioning (BRIEF)^f^, *Mdn* (*IQR*)				
Behavioral regulation index	40.0 (33.0–52.0)	50	< 0.001	0.59^c^
Metacognition index	42.0 (34.0–50.0)	50	< 0.001	0.59^c^
Total score	40.0 (33.0–50.0)	50	< 0.001	0.61^c^
Sleep behavior (CSHQ)^g^, *Mdn* (*IQR*)				
Total score	42.0 (37.0–46.0)	40.5	0.03	0.22^c^

Continuous variables are analyzed with one-sample *t* tests or one-sample Wilcoxon signed rank tests. Dichotomous variables are analyzed with chi-square tests. *p* < 0.05 is considered statistically significant for primary outcome analyses (CPI). *p* < 0.002 is considered statistically significant for secondary outcome analyses (Dunn–Šidák correction). Effect sizes are were calculated corresponding to each statistical method and interpreted according to Cohen’s guidelines [21]

^a^Effect size Cohen’s *d*, small = 0.2, medium = 0.5, large = 0.8

^b^Effect size phi, small = 0.1, medium = 0.3, large = 0.5

^c^Effect size Pearson’s *r*, small = 0.1, medium = 0.3, large = 0.5

^d^One atenolol-treated child had a missing CCI score, *n* = 104 (propranolol *n* = 36; atenolol *n* = 68)

^e^Results excluding two atenolol-treated outliers that deviated more than 3 SD from sample average due to unreliable assessment, *n* = 103 (propranolol *n* = 36; atenolol *n* = 67)

^f^Two atenolol-treated children had missing BRIEF scores, *n* = 103 (propranolol *n* = 36; atenolol *n* = 67)

^g^Four propranolol-treated children and two atenolol-treated children had missing CSHQ scores, *n* = 98 (propranolol *n* = 32; atenolol *n* = 66)

### Post hocanalysis: sex differences

Having included sex as a confounder in corrected analyses of the primary outcome measure, we found a significant association between sex and CPI scores, seemingly independent of beta-blocker type. Post hoc analyses showed that the CPI mean of males (*M* = 92.4, *SD* = 10.3) was 10.3 IQ points lower than the CPI mean of females (*M* = 102.7, *SD* = 14.7). Considering the small number of males (*n* = 20), we performed a Mann–Whitney *U* test to compare medians. The sex difference was significant (*p* = 0.001), with a medium effect size (*r* = 0.32). A sex difference with a small effect size (*r* = 0.25) was also observed for FSIQ (*p* = 0.009) but not for any other secondary outcomes (Supplementary Table S1). Sex differences were not found for variables such as age, mother’s education level, IH location, treatment type, dose, or treatment duration. To compare the sample of males more precisely with males from the general population, we obtained original Dutch normative data 1:1 matched by sex, mother’s education, and age from the authors of the WISC-V-NL. The CPI mean of males in the study sample was 12.4 IQ points lower than the CPI mean of matched males from the general population (*M* = 104.8, *SD* = 13.6), which was significant (Mann–Whitney *U* test, *p* = 0.003) with a medium effect size (*r* = 0.46).

## Discussion

This two-center study is the largest study to date investigating the long-term neurocognitive functioning of otherwise healthy children (age ≥ 6 years old) who, as infants, had received propranolol or atenolol for IH. Considering the drug characteristics of propranolol, we expected that children treated with propranolol would have lower scores on a pre-specified outcome measure for working memory, processing speed, and attention (CPI) in comparison to children treated with atenolol. Our results show no differences between the two groups for the CPI and secondary outcomes. Furthermore, neurocognitive outcomes did not differ between the total sample and children from the general population. However, in post hoc analyses, males had substantially lower CPI scores.

The finding that the level of neurocognitive functioning at school age was not different between children treated with either propranolol or atenolol during infancy is not in line with expectations based on the pharmacological characteristics and side effect profiles of these beta-blockers. Although propranolol passes the blood–brain barrier, it does not seem to affect neurocognitive development during infancy. If, nevertheless, any disruption of CNS development occurs under the influence of beta-blockers during infancy, the neurocognitive consequences of this disruption may be resolved by brain plasticity [24].

The lack of difference in the level of neurocognitive functioning between children treated with beta-blockers and children in the general Dutch population is in line with previous studies of children treated with propranolol for IH [11, 12]. These earlier studies, however, had small sample sizes, which limits the ability to draw conclusions. Our study analyzed 105 children and provides further evidence that beta-blockers are generally safe as far as long-term neurocognitive functioning is concerned. We found that scores on parent-reported executive functioning were better than norm scores. Since parents were aware of the research hypothesis, expectation bias may have influenced reporting. Nonetheless, the combined results illustrate that the studied children perform at an adequate level in both a research setting and daily life.

The results regarding the 20 males in our sample should be interpreted with caution. Males had substantially lower CPI scores, both compared to females treated with beta-blockers and compared to males from the normative sample (1:1 matched for sex, age, and mother’s education). This difference was considered clinically significant, as it may have implications for the educational attainment of these males [25]. In our sample, we found that females represented the full range of the normal distribution, whereas males only represented the lower range of the normal distribution. A similar distribution of scores was observed in raw data published by González-Llorente and colleagues (2017) [11]. Underlying mechanisms may be sex differences in brain plasticity and neurological vulnerability during infancy, pharmacokinetic differences between males and females, or unknown pathology leading to both IH and cognitive problems in males [26–28]. Given these results, we cannot be certain about the long-term safety of beta-blocker treatment in male infants until further research has been done with a larger sample. Working together, the clinician and parents should weigh the risks and benefits before starting treatment of IH with beta-blockers, especially when the child is male.

A strength of the current study is the substantial size of our unique cohort of children who received either propranolol or atenolol independent of disease characteristics. The large sample size enabled us to control for covariates such as sex, mother’s education level, and dose-related variables. Additionally, we applied measures that are sensitive to subtle deviations in neurocognitive functioning.

We maintained strict inclusion criteria. Therefore, the results cannot as yet be extrapolated to the entire population of children who have received beta-blockers for IH, e.g., preterm infants or children who have been treated for less than 6 months. Given our negative findings and in this strictly defined sample, we cannot exclude a type II error, although the CPI difference found between both beta-blocker groups is not considered clinically relevant. Additionally, as in previous research, the current research was limited by a lack of a suitable control group of children with complicated IH not receiving beta-blocker treatment, since withholding beta-blocker treatment for complicated IH is considered unethical.

In conclusion, this study provides robust evidence that for children with IH, treatment with propranolol or atenolol is not associated with long-term deficits in neurocognitive functioning. Although beta-blockers thus appear to be a safe treatment for IH with regard to long-term neurocognitive functioning, there are concerns about possible effects of this treatment on the long-term neurocognitive functioning of males.

## Supplementary Information

Below is the link to the electronic supplementary material.

Supplementary file1 (DOCX 44 KB)

## Abbreviations

ADHD: Attention-deficit/hyperactivity disorder
BRIEF: Behavioral Rating Inventory of Executive Functioning
CNS: Central nervous system
CPI: Cognitive Proficiency Index
CSHQ: Child Sleep Habits Questionnaire
Erasmus MC: Erasmus MC, University Medical Center Rotterdam, Rotterdam, the Netherlands
FSIQ: Full-scale intelligence quotient
GAI: General ability index
IH: Infantile hemangiomas
ISCED: International Standard Classification of Education
MD: Memory for Designs
MDD: Memory for Designs Delayed
NEPSY-II-NL: Developmental Neuropsychological Assessment-II, Dutch version
RAVLT: Rey Auditory Verbal Learning Test
UMCU: University Medical Center Utrecht, Utrecht, the Netherlands
WISC-V-NL: Wechsler Intelligence Scale for Children-V, Dutch version
