# Correction to: Long-term neurocognitive functioning of children treated with propranolol or atenolol for infantile hemangioma

**DOI:** 10.1007/s00431-023-05059-0

**Published:** 2023-06-26

**Authors:** Mireille M. Hermans, André B. Rietman, Renske Schappin, Peter C. J. de Laat, Elodie J. Mendels, Johannes M. P. J. Breur, Hester R. Langeveld, Saskia N. de Wildt, Corstiaan C. Breugem, Marlies de Graaf, Martine F. Raphael, Suzanne G. M. A. Pasmans

**Affiliations:** 1 Department of Dermatology, Erasmus MC Sophia Children’s Hospital, University Medical Center Rotterdam, Center of Pediatric Dermatology, Center of Rare Skin Diseases, Vascular Anomaly Center Erasmus MC Rotterdam, member of the ERN-SKIN-Mosaic group and ERN-VASCERN-VASCA group, Rotterdam, Netherlands; 2 Department of Child and Adolescent Psychology/Psychiatry, Erasmus MC Sophia Children’s Hospital, University Medical Center Rotterdam, Rotterdam, Netherlands; 3 Department of Surgery, Wilhelmina Children’s Hospital, University Medical Center Utrecht, Utrecht, Netherlands; 4 Department of Pediatrics(-Hemato-Oncology), Center of Rare Skin Diseases, Vascular Anomaly Center Erasmus MC Rotterdam, member of the ERN-SKIN-Mosaic group and ERN-VASCERN-VASCA group, Erasmus MC Sophia Children’s Hospital, University Medical Center Rotterdam, Rotterdam, Netherlands; 5 Department of Pediatric Cardiology, Wilhelmina Children’s Hospital, University Medical Center Utrecht, Utrecht, Netherlands; 6 Department of Intensive Care and Pediatric Surgery, Center of Rare Skin Diseases, Vascular Anomaly Center Erasmus MC Rotterdam, member of the ERN-SKIN-Mosaic group and ERN-VASCERN-VASCA group, Erasmus MC Sophia Children’s Hospital, University Medical Center Rotterdam, Rotterdam, Netherlands; 7 Department of Pharmacology and Toxicology, Radboud Institute for Health Sciences, Radboud University Medical Center, Nijmegen, Netherlands; 8 Department of Plastic Surgery, UMC Utrecht Center for Vascular Anomalies, Wilhelmina Children’s Hospital, University Medical Center Utrecht, Utrecht, Netherlands; 9 Department of Plastic, Reconstructive and Hand Surgery, UMC Location University of Amsterdam, Utrecht, Netherlands; 10 Department of Dermatology, UMC Utrecht Center for Vascular Anomalies, Wilhelmina Children’s Hospital, University Medical Center Utrecht, Utrecht, Netherlands; 11 Department Emma Children’s Hospital, UMC Location University of Amsterdam, Amsterdam, Netherlands

**Correction to: European Journal of Pediatrics (2022) 182:757–767** **https://doi.org/10.1007/s00431-022-04674-7**

In the original published version of the above article, the data reported with ‘yes’ and ‘no’ under "Child migration background" are switched up. The Table 1 below is the correct listing.

**Table 1:** Participant characteristics

	All (*n* = 105)	Propranolol (*n* = 36)	Atenolol (*n* = 69)	*p*-value
**Demographics**				
Child age, years				
Median (*IQR*)	7.4 (6.7–8.5)	8.0 (7.3–8.8)	7.1 (6.4–8.1)	< 0.001
Range	6.0–11.8	6.4–11.8	6.0–9.7	
Child sex, *n* (%)				
Female	85 (81)	29 (81)	56 (81)	> 0.99
Male	20 (19)	7 (19)	13 (19)	
Child migration background^a^, *n* (%)				
Yes	10 (10)	0 (0.0)	10 (14)	0.01
No	94 (90)	36 (100)	58 (84)	
Unknown	1 (1.0)	0 (0.0)	1 (1.4)	
Education mother, *n* (%)				
Low	14 (13)	6 (17)	8 (12)	0.89
Average	28 (27)	8 (22)	20 (29)	
High	62 (59)	22 (61)	40 (58)	
Unknown	1 (1.0)	0 (0.0)	1 (1.4)	
Home language, *n* (%)				
Dutch	97 (92)	35 (97)	62 (90)	0.37
Other	2 (2.0)	0 (0.0)	2 (2.9)	
Multilingual	6 (5.7)	1 (2.8)	5 (7.2)	
Confirmed diagnosis, *n* (%)				
Attention deficit/hyperactivity disorder	6 (5.7)	3 (8.3)	3 (4.3)	
**Clinical information**				
Location of IH^b^, *n* (%)				
Head and neck	84 (80)	24 (67)	60 (87)	0.02
Trunk	13 (12)	6 (17)	7 (10)	0.36
Genital area	13 (12)	7 (19)	6 (8.7)	0.13
Extremities	7 (6.7)	3 (8.3)	4 (5.8)	0.69
Ulcerated IH, *n* (%)				
Yes	29 (28)	13 (36)	16 (23)	0.16
No	76 (72)	23 (64)	53 (77)	
Treatment center, *n* (%)				
Erasmus MC	34 (32)	31 (86)	3 (4.3)	< 0.001
UMCU	71 (68)	5 (14)	66 (96)	
Age at treatment initiation, months				
Median (*IQR*)	3.5 (2.2–5.1)	3.6 (2.2–5.3)	3.4 (2.2–5.0)	0.58
Range	0.92–11.4	1.64–11.4	0.92–10.9	
Treatment duration, months				
Median (*IQR*)	13.8 (10.9–19.4)	18.6 (12.5–22.7)	13.0 (10.4–15.8)	0.001
Range	6.41–62.7	9.13–62.7	6.41–56.8	
Average dose, mg/kg/day				
Median (*IQR*)	1.2 (1.0–1.8)	1.9 (1.8–2.0)	1.0 (1.0–1.2)	< 0.001
Range	0.8–2.5	1.4–2.5	0.8–2.0	
Peak dose, mg/kg/day				
Median (*IQR*)	1.6 (1.0–2.1)	2.1 (2.0–2.3)	1.0 (1.0–1.6)	< 0.001
Range	1.0–14.0	1.9–14.0	1.0–3.0	
Cumulative dose, mg/kg				
Median (*IQR*)	577.4 (387.2–881.7)	1122.7 (718.6–1282.3)	418.7 (310.0–619.7)	< 0.001
Range	186.6–3544	494.1–3544	186.6–2206	
Follow-up time^c^, years				
Median (*IQR*)	5.9 (5.2–6.5)	6.2 (5.6–6.6)	5.7 (5.1–6.2)	0.13
Range	1.6–9.7	1.6–9.7	4.5–8.4	

*p* values indicate differences in participant characteristics between propranolol and atenolol group Continuous variables were not normally distributed and analyzed with a Mann-Whitney U test. Dichotomous variables were analyzed with a Fisher’s exact test

^a^Child migration background, categorized as “yes” = one or both parents born abroad or “no” = both parents born in the Netherlands

^b^A total of 105 patients had a total of 128 IH. The variable “location of IH” represents the number of children with at least one infantile hemangioma at each region

^c^Follow-up time: time interval between cessation of beta-blocker treatment and neuropsychological assessment

The original article has been corrected.

