# Supplementary material for: Long-term neurocognitive functioning of children treated with propranolol or atenolol for infantile hemangioma
Source: Eur J Pediatr. 2022 Dec 7;182(2):757–67. doi: 10.1007/s00431-022-04674-7 (PMC9899165; doi:10.1007/s00431-022-04674-7)
Supplement: Supplementary file 1 — Supplementary file1 (DOCX 44 KB) [file 431_2022_4674_MOESM1_ESM.docx]

Supplementary Appendix to manuscript entitled:

**Long-term neurocognition after treatment with propranolol or atenolol for infantile hemangioma**

*European Journal of Pediatrics*

Mireille M. Hermans MSc, André B. Rietman PhD & Renske Schappin PhD, Peter C. J. de Laat MD, PhD, Elodie J. Mendels MD, Johannes M. P. J. Breur MD, PhD, Hester R. Langeveld MD, PhD, Prof. Saskia N. de Wildt MD, PhD, Prof. Corstiaan C. Breugem MD, PhD, Marlies de Graaf MD, PhD, Martine F. Raphael MD, PhD, Prof. Suzanne G. M. A. Pasmans MD, PhD

Table of Contents

[Supplementary Methods: Systematic Literature Search 2](#_Toc71026555)

[Supplementary Methods: Statistical Analysis Plan 3](#_Toc71026556)

[Supplementary Results: Elaboration on Recruitment Procedures 5](#_Toc71026557)

[Supplementary Table S1 6](#_Toc71026566)

[Supplementary Table S2 7](#_Toc71026567)

[Supplementary References 8](#_Toc71026568)

# Supplementary Methods: Systematic Literature Search

embase.com:

('beta adrenergic receptor blocking agent'/de OR Atenolol/de OR Propranolol/de OR (((beta OR β) NEAR/3 (block* OR antoagonist*)) OR Atenolol* OR Propranolol*):ab,ti) AND (hemangioma/exp OR (hemangioma* OR haemangioma*):ab,ti) AND (neurocognition/exp OR attention/exp OR 'working memory'/de OR intelligence/de OR 'processing speed'/de OR 'nerve cell differentiation'/de OR 'neuropsychological test'/de OR psychology/exp OR 'mental disease'/exp OR 'mental function'/de OR cognition/exp OR personality/exp OR psycholinguistics/exp OR psychophysiology/de OR sleep/exp OR 'executive function'/exp OR 'sleep disorder'/exp OR 'psychomotor performance'/de OR 'psychomotor activity'/de OR 'academic achievement'/exp OR 'central nervous system function'/de OR (neurocogniti* OR attention* OR memor* OR intelligence OR processing-speed* OR Neurodevelopment* OR neuropsycholog* OR psycholog* OR mental* OR learning OR cogniti* OR personalit* OR psychophysiolog* OR sleep OR (executive NEAR/3 function*) OR psychomotor* OR ((academic* OR school*) NEAR/3 (achievement* OR performance* OR failure OR grade* OR success)) OR ((central-nervous-system* OR cns OR brain OR mental) NEAR/3 function*)):Ab,ti) NOT [conference abstract]/lim

Medline ALL Ovid:

(Adrenergic beta-Antagonists/ OR Atenolol/ OR Propranolol/ OR (((beta) ADJ3 (block* OR antoagonist*)) OR Atenolol* OR Propranolol*).ab,ti.) AND (exp Hemangioma/ OR (hemangioma* OR haemangioma*).ab,ti.) AND (exp Neurocognitive Disorders/ OR exp Cognitive Dysfunction/ OR Attention/ OR Memory, Short-Term/ OR exp Intelligence/ OR Neuropsychological Tests/ OR exp Psychology/ OR exp Mental Disorders/ OR exp Cognition/ OR exp Personality/ OR Psycholinguistics/ OR exp Psychophysiology/ OR exp Sleep/ OR Executive Function/ OR exp Sleep Wake Disorders/ OR Psychomotor Performance/ OR exp Academic Success/ OR (neurocogniti* OR attention* OR memor* OR intelligence OR processing-speed* OR Neurodevelopment* OR neuropsycholog* OR psycholog* OR mental* OR learning OR cogniti* OR personalit* OR psychophysiolog* OR sleep OR (executive ADJ3 function*) OR psychomotor* OR ((academic* OR school*) ADJ3 (achievement* OR performance* OR failure OR grade* OR success)) OR ((central-nervous-system* OR cns OR brain OR mental) ADJ3 function*)).ab,ti.)

PsycINFO Ovid:

(Adrenergic beta-Antagonists / OR Atenolol/ OR Propranolol/ OR (((beta) ADJ3 (block* OR antoagonist*)) OR Atenolol* OR Propranolol*).ab,ti.) AND (exp Hemangioma/ OR (hemangioma* OR haemangioma*).ab,ti.) AND (exp Neurocognitive Disorders/ OR exp Cognitive Dysfunction/ OR Attention/ OR Memory, Short-Term/ OR exp Intelligence/ OR Neuropsychological Tests/ OR exp Psychology/ OR exp Mental Disorders/ OR exp Cognition/ OR exp Personality/ OR Psycholinguistics/ OR exp Psychophysiology/ OR exp Sleep/ OR Executive Function/ OR exp Sleep Wake Disorders/ OR Psychomotor Performance/ OR exp Academic Success/ OR (neurocogniti* OR attention* OR memor* OR intelligence OR processing-speed* OR Neurodevelopment* OR neuropsycholog* OR psycholog* OR mental* OR learning OR cogniti* OR personalit* OR psychophysiolog* OR sleep OR (executive ADJ3 function*) OR psychomotor* OR ((academic* OR school*) ADJ3 (achievement* OR performance* OR failure OR grade* OR success)) OR ((central-nervous-system* OR cns OR brain OR mental) ADJ3 function*)).ab,ti.)

Web of Science Core Collection:

TS=(((((beta OR β) NEAR/2 (block* OR antoagonist*)) OR Atenolol* OR Propranolol*)) AND ((hemangioma* OR haemangioma*)) AND ((neurocogniti* OR attention* OR memor* OR intelligence OR processing-speed* OR Neurodevelopment* OR neuropsycholog* OR psycholog* OR mental* OR learning OR cogniti* OR personalit* OR psychophysiolog* OR sleep OR (executive NEAR/2 function*) OR psychomotor* OR ((academic* OR school*) NEAR/2 (achievement* OR performance* OR failure OR grade* OR success)) OR ((central-nervous-system* OR cns OR brain OR mental) NEAR/2 function*))))

Cochrane CENTRAL register of Trials:

((((beta OR β) NEAR/3 (block* OR antoagonist*)) OR Atenolol* OR Propranolol*):ab,ti) AND ((hemangioma* OR haemangioma*):ab,ti) AND ((neurocogniti* OR attention* OR memor* OR intelligence OR processing NEXT speed* OR Neurodevelopment* OR neuropsycholog* OR psycholog* OR mental* OR learning OR cogniti* OR personalit* OR psychophysiolog* OR sleep OR (executive NEAR/3 function*) OR psychomotor* OR ((academic* OR school*) NEAR/3 (achievement* OR performance* OR failure OR grade* OR success)) OR ((central NEXT nervous NEXT system* OR cns OR brain OR mental) NEAR/3 function*)):Ab,ti)

# Supplementary Methods: Statistical Analysis Plan

Pre-analysis:

- Data from the neuropsychological assessments are collected and scored on paper source documents and then entered into the online OpenClinica database. Data obtained from patient records are entered into the SPSS database.
- All data collected from the first three patients plus 1/10 randomly selected patients will be completely checked for accuracy. This applies to both paper scoring and data entry into the OpenClinica or SPSS database. If any systematic errors are observed within one variable, the data of all participants concerning that variable will be checked and (if necessary) corrected. Both the investigator involved in the initial data scoring and entry process, and an investigator not involved in the initial data scoring and entry process, individually perform such a data quality check.
- If a patient meets one of the exclusion criteria, the patient will not be included in the data analysis.
- Handling of missing data: there will be no replacement of missing data concerning the dependent variable (complete case analysis). If any missings occur in any of our covariates, we will consider multiple imputations or complete case analysis based on whether the data is missing completely at random. We follow the guidelines established by Jakobsen, Gluud, Wetterslev, & Winkel (2017)[1] and White & Carlin (2010)[2].
- All test assumptions will be checked prior to data analysis.
- Checking for assumptions such as normality, both clinical groups separately, using inspection of plots, mean/median, kurtosis/skewness, and Shapiro-Wilk-testing.
- Comparing differences of demographics and disease characteristics between both clinical groups.
- Correction for multiple comparisons: Dunn-Šidák correction.

Primary research question

**Is there a difference in long-term neurocognitive functioning between children treated with propranolol or atenolol in the first year of life for infantile hemangioma?**

Dependent: Cognitive Proficiency Index (CPI) of the WISC-V-NL (interval, IQ scale with mean 100, SD 15)

Independent: beta-blocker type

Alpha: .05; two-sided

Test: When CPI scores are normally distributed, we will perform an independent samples t-test.

We expect the CPI to be normally distributed, because beta-blockers may negatively influence the CPI, but will not be determinative for the CPI. Therefore, we expect a shift in the normal distribution to the left and not a skewed distribution.

If CPI scores are not normally distributed, we will perform a Mann-Whitney U test.

To control for covariate effects, we will perform a multivariable linear regression analysis with the CPI as dependent variable and beta-blocker, child’s sex, mother’s education, cumulative dose, treatment duration, and age at treatment initiation as independent variables. If the residuals of the CPI are not normally distributed, we will perform a transformation of the dependent variable.

Secondary research questions

**Is there a difference in long-term neurocognitive functioning between children treated with propranolol or atenolol and the test norm group?**

Dependent: Cognitive Proficiency Index (CPI) of the WISC-V-NL (interval, IQ scale with mean 100, SD 15))

Independent: beta-blocker use and/or type

Alpha: .05

The analysis depends on the outcome of the primary research question:

- If the CPI for atenolol and propranolol is significantly different, we will perform two one-sample t-tests to investigate whether the mean CPI differs from 100 (the population mean).
- If the CPI for atenolol and propranolol is not significantly different, we will combine the beta-blocker groups and perform one one-sample t-test to investigate whether the mean CPI of all children that used beta-blockers differs from 100 (the population mean).

**For all secondary outcomes: is there a difference between the atenolol and propranolol group and the population norm.**

For the secondary outcomes of interval level, the analyses described under the first two questions are repeated.

To analyze the difference between the propranolol and atenolol group, secondary outcomes at ordinal level are analyzed with Mann-Whitney U-tests and secondary outcomes at nominal level are analyzed with a Fisher’s exact test. When ordinal population norm data is available, we will use chi square test to analyze the difference between the beta-blocker group(s) and the population norm.

# **Supplementary Results: Elaboration on Recruitment Procedures**

As addressed in Figure 1, 299 patient records were screened for inclusion criteria. Initially, 162 children were deemed eligible (54% of patient records). Their parent(s)/legal guardian(s) were contacted to inquire about willingness to participate in the study. Parent(s)/legal guardian(s) of 29 children refused to participate (48% treated with propranolol; 90% female). Out of these 29 parents/legal guardians, 16 elaborated on their child’s health status. Seven parents (44%) noted their child was having one or more health issues, including emotional and behavioral problems (*n*=3), cognitive impairment (*n*=1), cognitive impairment and metabolic disease (*n*=1), immunological problems (*n*=1), and unexplained abdominal pain (*n*=1). The other 9 parents (56%) certified their child was not having any health issues at all.

|  | Propranolol | Atenolol |
| --- | --- | --- |
| Health issues | *n*=5 | *n*=2 |
| No health issues | *n*=4 | *n*=5 |

# **Supplementary Table S1**

Supplementary Table 1. Descriptive statistics of males and females treated with beta-blockers for infantile hemangioma, and corresponding normative sample matched for child age, child sex, and mother’s education

|  | Sample males  (*n=*20) | Matched norm males | Sample females  (*n=*85) | Matched norm females |
| --- | --- | --- | --- | --- |
| Intelligence (WISC-V-NL), *M* (*SD*) |  |  |  |  |
| **Cognitive Proficiency Index**^1^ | 92.4 (10.3) | 104.8 (13.6) | 102.7 (14.7) | 106.1 (13.7) |
| General Ability Index | 97.7 (11.0) | 102.7 (13.4) | 102.3 (12.1) | 104.1 (12.8) |
| Full Scale IQ | 94.8 (10.1) | 103.3 (12.9) | 102.1 (12.5) | 105.1 (12.8) |
| Visual Spatial Memory (NEPSY-II-NL), *n* (%) |  |  |  |  |
| Immediate recall |  |  |  |  |
| Clinical range (pct ≤ 10) | 1 (5.0) | N.A. | 14 (16) | N.A. |
| Non-clinical range (pct > 10) | 19 (95) | N.A. | 71 (84) | N.A. |
| Delayed recall |  |  |  |  |
| Clinical range (pct ≤ 10) | 4 (20) | N.A. | 11 (13) | N.A. |
| Non-clinical range (pct > 10) | 16 (80 | N.A. | 74 (87) | N.A. |
| Narrative Memory (NEPSY-II-NL), *n* (%) |  |  |  |  |
| Clinical range (pct ≤ 10) | 3 (15) | N.A. | 7 (8.2) | N.A. |
| Non-clinical range (pct > 10) | 17 (85) | N.A. | 78 (92) | N.A. |
| Auditory Memory (RAVLT)^2^, *M* (*SD*) |  |  |  |  |
| Immediate recall | -0.4 (1.0) | N.A. | -0.3 (1.1) | N.A. |
| Delayed recall | -0.4 (0.9) | N.A. | -0.3 (1.1) | N.A. |
| Executive Functioning (BRIEF)^3^, *Mdn* (*IQR*) |  |  |  |  |
| Behavioral Regulation Index | 40.5 (34.0 – 52.0) | N.A. | 40.0 (33.0 – 52.0) | N.A. |
| Metacognition Index | 42.5 (33.0 – 50.0) | N.A. | 41.0 (34.0 – 51.0) | N.A. |
| Total score | 41.5 (31.0 – 47.5) | N.A. | 40.0 (33.0 – 51.0) | N.A. |
| Sleep behavior (CSHQ)^4^, *Mdn* (*IQR*) |  |  |  |  |
| Total score | 40.0 (37.0 – 46.0) | N.A. | 42.0 (37.0 – 46.0) | N.A. |

N.A., not applicable.

^1^One atenolol-treated female had a missing CCI score, *n=*104 (propranolol *n=*36; atenolol *n=*68).
^2^Results excluding two atenolol-treated outliers (both females) that deviated more than 3 SD from sample average due to unreliable assessment, *n*=103 (propranolol *n=*36; atenolol *n=*67).
^3^Two atenolol-treated children (both females) had missing BRIEF scores, *n=*103 (propranolol *n=*36; atenolol *n=*67).
^4^Four propranolol-treated children (three females, one male) and two atenolol-treated children (both females) had missing CSHQ scores, *n=*98 (propranolol *n=*32; atenolol *n=*66).

# Supplementary Table S2

Supplementary Table S2. Characteristics of males and females treated with beta-blockers (propranolol or atenolol) for infantile hemangioma

|  | Males (*n=*20) | Females (*n=*85) |
| --- | --- | --- |
| **Demographics** | | |
| Child age, years |  |  |
| Median (*IQR*) | 7.5 (6.9 – 8.0) | 7.4 (6.5 – 8.2) |
| Range | 6.0 – 10.9 | 6.5 – 11.8 |
| Child migration background^1^, *n* (%) |  |  |
| Yes | 3 (15) | 7 (8.2) |
| No | 17 (85) | 77 (91) |
| Unknown | 0 (0.0) | 1 (1) |
| Education mother, *n* (%) |  |  |
| Low | 3 (15) | 11 (13) |
| Average | 7 (35) | 21 (25) |
| High | 10 (50) | 52 (61) |
| Unknown | 0 (0.0) | 1 (1.2) |
| Home language, *n* (%) |  |  |
| Dutch | 17 (85) | 81 (95) |
| Other | 0 (0.0) | 1 (1.2) |
| Multilingual | 3 (15) | 3 (3.5) |
| Confirmed diagnosis, *n* (%) |  |  |
| Attention deficit/hyperactivity disorder | 1 (5.0) | 5 (5.9) |
| **Clinical information** | | |
| Location of IH^2^, *n* (%) |  |  |
| Head and neck region | 18 (90) | 66 (78) |
| Trunk | 1 (5.0) | 12 (14) |
| Genital area | 2 (10) | 11 (13) |
| Extremities | 0 (0.0) | 7 (8.2) |
| Ulcerated IH, *n* (%) |  |  |
| Yes | 6 (30) | 23 (27) |
| No | 14 (70) | 62 (73) |
| Beta-blocker treatment, *n* (%) |  |  |
| Propranolol | 7 (35) | 29 (34) |
| Atenolol | 13 (65) | 56 (66) |
| Treatment center, *n* (%) |  |  |
| Erasmus MC | 6 (30) | 28 (33) |
| UMCU | 14 (70) | 57 (67) |
| Age at treatment initiation, months |  |  |
| Median (*IQR*) | 3.6 (2.6 – 5.7) | 3.5 (2.1 – 5.1) |
| Range | 1.7 – 11.4 | 0.9 – 8.3 |
| Treatment duration, months |  |  |
| Median (*IQR*) | 13.6 (10.2 – 19.5) | 13.7 (11.2 – 19.2) |
| Range | 7.5 – 41.3 | 6.4 – 62.7 |

Supplementary Table S2. Characteristics of males and females treated with beta-blockers (propranolol or atenolol) for infantile hemangioma (continued)

|  | Males (*n=*20) | Females (*n=*85) |
| --- | --- | --- |
| Average dose, mg/kg/day |  |  |
| Median (IQR) | 1.5 (1.0 – 1.8) | 1.2 (1.0 – 1.8) |
| Range | 0.9 – 2.1 | 0.8 – 2.5 |
| Peak dose, mg/kg/day |  |  |
| Median (*IQR*) | 2.0 (1.1 – 2.1) | 1.5 (1.0 – 2.1) |
| Range | 1.0 – 2.7 | 1.0 – 14.0 |
| Cumulative dose, mg/kg |  |  |
| Median (*IQR*) | 624.5 (337.8 – 1099.7) | 562.1 (387.2 – 846.6) |
| Range | 214.5 – 2206.1 | 186.6 – 3544.0 |

^1^Child migration background, categorized as: ‘yes’ = one or both parents born abroad, or ‘no’ = both parents born in the Netherlands.
^2^The variable ‘location of IH’ encompasses all IH involved in the study. In the current study, a total of 105 patients had a total of 128 IH.

# Supplementary References

1. Jakobsen JC, Gluud C, Wetterslev J, Winkel P (2017) When and how should multiple imputation be used for handling missing data in randomised clinical trials – a practical guide with flowcharts. BMC Med Res Methodol 17:162

2. White IR, Carlin JB (2010) Bias and efficiency of multiple imputation compared with complete-case analysis for missing covariate values. Stat Med 29:2920-2931
